# TgPRELID, a Mitochondrial Protein Linked to Multidrug Resistance in the Parasite *Toxoplasma gondii*

**DOI:** 10.1128/mSphere.00229-16

**Published:** 2017-02-01

**Authors:** Victoria Jeffers, Edwin T. Kamau, Ananth R. Srinivasan, Jonathan Harper, Preethi Sankaran, Sarah E. Post, Joseph M. Varberg, William J. Sullivan, Jon P. Boyle

**Affiliations:** a Department of Pharmacology and Toxicology, Indiana University School of Medicine, Indianapolis, Indiana, USA; b Department of Biological Sciences, University of Pittsburgh, Pittsburgh, Pennsylvania, USA; c Department of Microbiology and Immunology, Indiana University School of Medicine, Indianapolis, Indiana, USA; University of Texas Southwestern

**Keywords:** PRELI domain, *Toxoplasma gondii*, mitochondrial protein import, multidrug resistance

## Abstract

New drugs to control infection with the protozoan parasite *Toxoplasma gondii* are needed as current treatments exert toxic side effects on patients. Approaches to develop novel compounds for drug development include screening of compound libraries and targeted inhibition of essential cellular pathways. We identified two distinct compounds that display inhibitory activity against the parasite’s replicative stage: F3215-0002, which we previously identified during a compound library screen, and I-BET151, an inhibitor of bromodomains, the “reader” module of acetylated lysines. In independent studies, we sought to determine the targets of these two compounds using forward genetics, generating resistant mutants and identifying the determinants of resistance with comparative genome sequencing. Despite the dissimilarity of the two compounds, we recovered resistant mutants with nonsynonymous mutations in the same domain of the same gene, *TGGT1_254250*, which we found encodes a protein that localizes to the parasite mitochondrion (designated TgPRELID after the name of said domain). We found that mutants selected with one compound were cross resistant to the other compound, suggesting a common mechanism of resistance. To further support our hypothesis that *TgPRELID* mutations facilitate resistance to both I-BET151 and F3215-0002, CRISPR (clustered regularly interspaced short palindromic repeat)/CAS9-mediated mutation of *TgPRELID* directly led to increased F3215-0002 resistance. Finally, all resistance mutations clustered in the same subdomain of TgPRELID. These findings suggest that *TgPRELID* may encode a multidrug resistance factor or that I-BET151 and F3215-0002 have the same target(s) despite their distinct chemical structures.

**IMPORTANCE** We report the discovery of TgPRELID, a previously uncharacterized mitochondrial protein linked to multidrug resistance in the parasite *Toxoplasma gondii*. Drug resistance remains a major problem in the battle against parasitic infection, and understanding how TgPRELID mutations augment resistance to multiple, distinct compounds will reveal needed insights into the development of new therapies for toxoplasmosis and other related parasitic diseases.

## Abstract (precis)

We report the discovery of TgPRELID, a previously uncharacterized mitochondrial protein linked to multidrug resistance in the parasite *Toxoplasma gondii*. Drug resistance remains a major problem in the battle against parasitic infection, and understanding how TgPRELID mutations augment resistance to multiple, distinct compounds will reveal needed insights into the development of new therapies for toxoplasmosis and other related parasitic diseases.

## INTRODUCTION

*Toxoplasma gondii* is a globally ubiquitous pathogen that infects ~20% of the world population. This obligate intracellular parasite is found in virtually all warm-blooded vertebrates, with feline species serving as the definitive host. An important feature of *Toxoplasma* infection is that the parasite persists for the lifetime of the host as a latent tissue cyst, and there is no available treatment that can cure an infected individual. Current drug therapies primarily target the rapidly growing tachyzoite life stage, but chronic cyst stages (which persist in the central nervous system [CNS] and muscle tissue) are refractory to all approved therapies. The persistence of *Toxoplasma* in the form of tissue cysts presents a risk of recurrent reactivated infection, which typically occurs in immunocompromised patients. The need for repeated drug courses coupled with the toxic side effects of the front-line antifolates highlights the need for better, safer treatments to be developed.

F3215-0002 (referred to here as F0002) is a 3,6-disubstituted triazole/thiadiazole-containing compound that was previously identified in a screen for compounds that inhibited *Toxoplasma* growth *in vitro* (1). The ~500 screened compounds were selected informatically based on their potential to have kinase-targeting ability. F0002 has a 50% inhibitory concentration (IC_50_) against parasite growth of ~1 μM; this effect is reversible, and its main target is the parasite rather than the host cell (1). The fact that a triazole/thiadiazole-containing compound has antimicrobial effects is not surprising, as this class has been found to have broad efficacy against a variety of bacterial and fungal pathogens (2–5). In certain cases, compounds such as these have been found to directly target kinases (2), but the direct target(s) of F0002 and the other inhibitory compounds is currently unknown.

The posttranslational modification of proteins by lysine acetylation has previously been validated as a drug target in *Toxoplasma*, whereby chemical inhibition of either the enzymes that add the acetyl mark (lysine acetyltransferases) or those that remove the acetyl mark (lysine deacetylases) leads to parasite death (6; also reviewed in reference 7). However, to date, no studies have evaluated the effect of bromodomain inhibitors on *Toxoplasma* proliferation. The bromodomain is considered the “reader” domain of acetylated lysine residues; its structure forms a hydrophobic pocket that fits the acetylated lysine and facilitates recruitment of gene regulatory factors or other downstream signaling factors (8). One of the first bromodomain inhibitors to be described is I-BET151, which inhibits the bromodomain and extraterminal (BET) family of bromodomain proteins and was initially found to modulate the inflammatory response to bacterial lipopolysaccharide through dysregulation of inflammatory cytokines and chemokines (9). I-BET151 has already shown promise as a potential antiparasitic in that treatment of infection with the African sleeping sickness parasite *Trypanosoma brucei* leads to disruption of stage-specific genes as well as dysregulation of the variable surface glycoproteins that are critical for immune evasion (10).

In the present study, the Boyle and Sullivan laboratories were independently employing forward genetic approaches to identify candidate molecular targets for these two compounds, which belong to divergent compound classes. Using ethylnitrosourea (ENU)-based mutagenesis, drug selection, and next-generation sequencing, we unexpectedly found that mutations in the same domain (PRELI) of the same gene (*TGGT1_254250*; *TgPRELID*) are associated with resistance to both of these anti-*Toxoplasma* compounds. The TgPRELID protein, which we also localized to the parasite mitochondrion, represents the first description of a PRELI domain protein in apicomplexan parasites. Mutants selected for their resistance to one compound are resistant to the other, indicating that TgPRELID may play a role in multidrug resistance in *Toxoplasma* and possibly other parasites in the phylum Apicomplexa.

## RESULTS

### *Toxoplasma* mutants highly resistant to F0002 show no growth defects.

**FIG 1:**
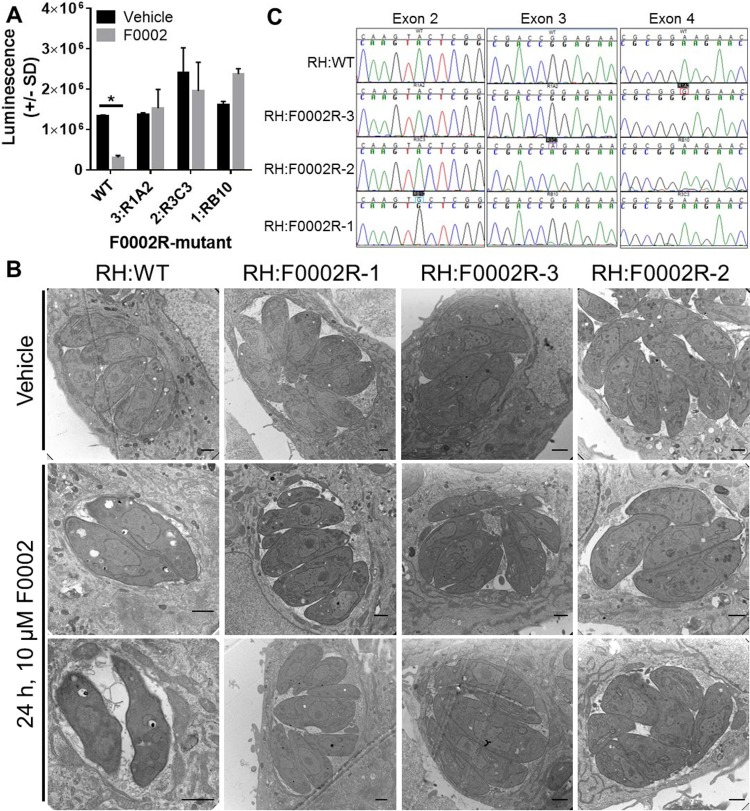
(A) Sensitivity of WT *Toxoplasma* and F0002R mutants 1, 2, and 3 to 10 μM F0002. Growth was determined using bioluminescence. Following 2-way ANOVA, all means were compared to WT with vehicle using 2-way ANOVA followed by *post hoc* tests as described in Materials and Methods. *, *P* < 0.05 compared to WT with vehicle. *n =* 2 wells per strain-drug combination. (B) Ultrastructure of F0002-resistant mutants and wild-type parasites in the presence of 10 μM F0002 or vehicle for 24 h. Bars, 1 μm. (C) Sanger sequence validation of SNPs in F0002R mutants 1, 2, and 3.

We previously identified compound F0002 in a screen for compounds that inhibit *Toxoplasma* tachyzoite replication. To identify targets of F0002, we obtained parasites that were resistant to F0002 in three separate ENU experiments and isolated one clone from each. We named these three clones 1RB10, 2R3C3, and 3R1A2 and tested their overall resistance to 10 μM F0002. Based on luciferase-based growth assays, all three mutants were highly resistant to F0002, growing at a similar rate as vehicle-treated parasites (*P* > 0.05) (Fig. 1A). In contrast, the wild-type (WT) strain (RH:WT) was susceptible to F0002 as expected (*P* = 0.02 compared to vehicle treatment). This difference in susceptibility is further illustrated in electron micrographs of all 3 mutant lines (Fig. 1B). While wild-type *T. gondii* exhibited clear growth restriction and vacuolar disruption after 24 h of exposure to 10 μM F0002, mutant parasites replicated and were found within intact vacuoles after drug exposure.

### *TGGT1_254250* is mutated in three genetically distinct F0002R mutants.

**FIG 2:**
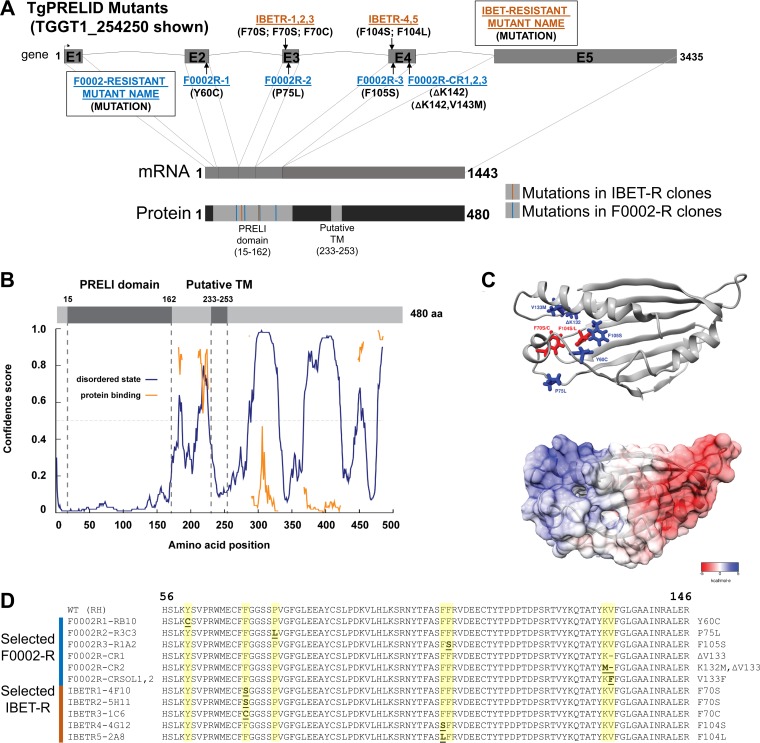
(A) Schematic of the *TGGT1_254250/TgPRELID* gene, mRNA, and predicted protein. Intron-exon boundaries were predicted in ToxoDB and confirmed by direct sequencing of TGGT1_254250 transcript. Protein domain searches were done using Pfam, identifying a PRELI domain in amino acid residues 15 to 162. A putative transmembrane domain was also predicted using the Psipred and TMPred servers (residues 233 to 253). (B) Disopred3 analysis of TgPRELID polypeptide indicates a high probability of order in the first 150 amino acids (aa) that corresponds to the PRELI domain. Extensive patches of disorder are predicted C terminal to the PRELI domain. Some of these are also predicted to be protein binding (orange trace). (C) I-TASSER-predicted structure of TgPRELID PRELI domain (C score = 1.18), with identified mutations highlighted (IBETR, red; F0002R, blue). The electrostatic surface potential was calculated using APBS and mapped to the TgPRELID surface, in a gradient from negatively charged residues (red) to positively charged residues (blue). (D) Location of all TgPRELID mutations identified in ENU and CRISPR screens for F0002- and I-BET151-resistant mutants. All mutations associated with resistance to either F0002 or I-BET151 were located within the putative PRELI domain. In addition to F0002R mutants 1, 2, and 3, the three classes of CRISPR/CAS9-mediated resistance mutations are shown (CR1 and CR2 and that found in clones CRSOL1 and CRSOL2). All CRISPR-driven mutations showed no evidence for incorporation of the homology repair template containing the F105S mutation but instead resulted in either the deletion or the mutation of valine 133.

We sequenced the entire genomes of three F0002-resistant (F0002R) mutants and the wild-type parental strain to coverage levels ranging from 37× to 73× (Table 1). After bioinformatic single nucleotide polymorphism (SNP) curation, we identified 21, 20, and 33 SNPs, respectively. Upon manual inspection, we curated this list down to 19 total SNPs that were found in coding regions (8, 8, and 3 across the three F0002-resistant mutants, respectively). Of these 19 polymorphisms, 17 produced nonsynonymous mutations spanning 17 distinct genes. Only one gene, *TGGT1_254250* (v7.3 identifier [ID], *TGGT1_003655*), had a mutation in all three of the F0002-resistant mutants, and each of these three mutations was a nonsynonymous mutation. These SNPs were validated in each clone by Sanger sequencing of PCR amplicons (Fig. 1C) and mapped to the predicted intron-exon structure of *TGGT1_254250* (Fig. 2A). Therefore, *TGGT1_254250* became the top candidate gene for conferring F0002 resistance. The predicted TGGT1_254250 protein is annotated at ToxoDB (http://toxodb.org/toxo/) as having a PRELI domain in its N terminus (Fig. 2A to C); in yeast, proteins with this domain are responsible for phospholipid import to the inner mitochondrial membrane (11, 12). Consistent with the annotation, the only identified domain in TGGT1_254250 is the PRELI-like family domain (PF04707) between residues 15 and 172 (Fig. 2A to C). All three mutations linked to F0002 resistance were found in a small region of the predicted PRELI domain (Fig. 2A to D). Since TGGT1_254250 was predicted to be 482 amino acids long, we analyzed the entire protein using other domain-finding software programs, including Psipred and Disopred3. Interestingly, multiple regions immediately following the PRELI domain are predicted to be highly disordered and are interspersed with shorter putatively ordered regions (Fig. 2B). Of particular note is a region predicted to be ordered between positions 232 and 289, which is immediately preceded and followed by regions of predicted disorder. This ordered region corresponds with a putative transmembrane (TM) domain in TGGT1_254250 between positions 233 and 253 (Fig. 2A and B; also see Fig. S1A in the supplemental material). Lacking any other functional protein domains, we named the TGGT1_254250 gene “*TgPRELID*.” A survey of the available apicomplexan genome sequences on EuPathDB identified TgPRELID homologues in other coccidians, *Hammondia hammondi*, *Neospora caninum*, *Sarcocystis* spp., and *Eimeria* spp. A *TgPRELID* homologue was similarly found in *Plasmodium* spp.; however, the *Plasmodium* homologues are shorter due to the lack of an extended C-terminal region that is found only in the coccidian parasites (Fig. S1A and B). This suggests a functional split in PRELID proteins in coccidians and *Plasmodium* spp. This split is further illustrated by the neighbor-joining tree of coccidian and *Plasmodium* orthologues based on an alignment of the PRELI domain (the pink highlighted residues in Fig. S1A and the tree in S1B).

10.1128/mSphere.00229-16.1 FIG S1  (A) Multiple sequence alignment of TgPRELID orthologues from other coccidians, *Eimeria* spp., and *Plasmodium* spp. Orthologues were identified using BLASTP on ToxoDB and aligned using Clustal Omega. The PRELI domain was identified in all orthologues and is highlighted in pink. Percent conservation across all orthologues is indicated in different shades of purple (residues are colored purple if they agree with at least 50% of the residues in the column). The tentative TM (as identified by TMPred and Psipred) is indicated in light blue. While all orthologues share a highly significant (*P* ≥ 8 × 10^−9^) match to the PRELI domain (PF04707), only the coccidian parasites shown have the putatively disordered C-terminal extension. Moreover, only the tissue-dwelling coccidian parasites (*Toxoplasma*, *Hammondia*, *Neospora*, and *Sarcocystis*) have evidence for a putative TM in the mostly disordered C terminus. See Fig. 2B for a map of predicted protein disorder across the TgPRELID predicted protein. The three dots after the third sequence block indicate sequence alignment that was removed for clarity. (B) Neighbor-joining tree of TgPRELID orthologues aligned in panel A measured across the conserved PRELI domain (residues colored in pink). Scale bar represents 3 amino acid substitutions per 100 residues. Download FIG S1, DOCX file, 1.4 MB. Copyright © 2017 Jeffers et al. 2017 Jeffers et al. This content is distributed under the terms of the Creative Commons Attribution 4.0 International license.

**TABLE 1:** Raw sequence data and SNP identification in F0002-resistant mutants

Strain	WT	F0002R1-RB10	F0002R3-R1A2	F0002R2-R3C3
Total no. of reads				
Paired	77,943,132	74,463,104	77,445,016	53,393,440
Mapping	71,910,632	66,953,767	65,902,433	34,393,334
Avg coverage	73×	68×	68×	37×
No. of SNPs				
Candidate	NA^a^	21	20	33
Curated	NA	8	8	3
Curated (nonsynonymous)	NA	6	8	3

a NA, not applicable.

### Bromodomain inhibitor I-BET151 inhibits *Toxoplasma* replication, and TgPRELID is associated with resistance.

**FIG 3:**
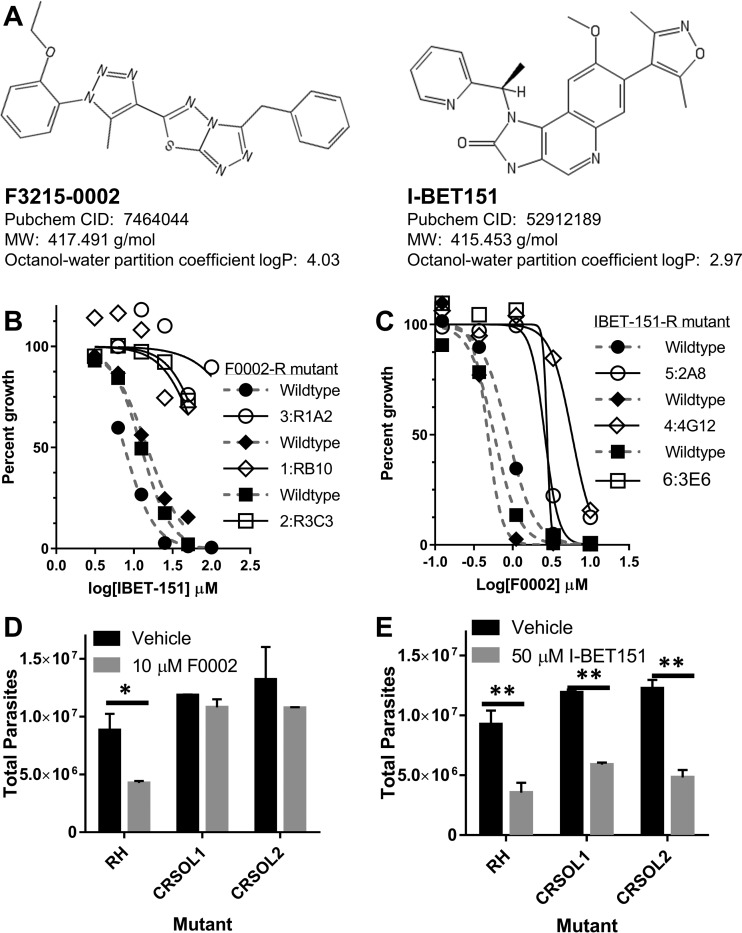
Quantification of drug cross-resistance between mutants selected for I-BET151 resistance and F0002 resistance. (A) Chemical structures of F3215-0002 (F0002) and I-BET151. PubChem identifiers and other properties relevant to their drug-like properties are listed. (B) Three F0002-resistant mutants were challenged in *in vitro* growth assays with 3.13 to 100 μM I-BET151, and the effect on parasite growth was assessed by direct parasite counting. All 3 mutants shown had significantly different dose-response curves (Table 2) and increases in IC_50_ values compared to wild-type assays performed in parallel (*P* < 0.05) (Table 2). All wild-type (filled shapes, dashed gray lines) and mutant (open shapes, black lines) fitted curves are shown and are paired by shape. *n =* 1 for each drug concentration for each mutant. IC_50_ and *P* values are listed in Table 2. (C) Six I-BET151-resistant mutants were challenged in identical assays with 0.123 to 10 μM F0002, and the effect on parasite growth was assessed by direct parasite counting. The 3 mutants shown (out of 6 total) had significantly different dose-response curves (*P* < 0.05) (Table 2) compared to wild type. Shape, line, and color are as in panel B. *n =* 1 for each drug concentration for each mutant. IC_50_ and *P* values are listed in Table 2. (D and E) Two *TgPRELID* F0002-resistant mutant clones generated by CRISPR/CAS9 and nonhomologous end-joining DNA repair were further tested for their resistance to 50 μM I-BET151 (E) and 10 μM F0002 (D) by direct parasite counting 24 h after drug exposure. Following 2-way ANOVA, all values were compared to RH with vehicle using *post hoc* tests as described in Materials and Methods. *, *P* < 0.05; **, *P* < 0.01, compared to RH with vehicle. *n =* 2 wells per strain-drug combination.

An independent search for novel inhibitors of *Toxoplasma* examined the possibility of targeting bromodomains. The bromodomain is the “reader” module of acetylated lysines, binding the acetylated lysine and initiating downstream signaling through recruitment of other proteins (13). Our previous studies have shown that there are hundreds of acetylated proteins in the *Toxoplasma* proteome, potentially regulating many diverse cellular functions (14, 15). The parasite genome contains 12 bromodomain-containing proteins, including two previously characterized GCN5-family lysine acetyltransferases that we have shown previously to be critical for parasite viability and differentiation (16, 17). Therefore, we investigated the effect of the bromodomain inhibitor I-BET151 (Fig. 3A) on *Toxoplasma* and determined that I-BET151 inhibited tachyzoite proliferation with an IC_50_ of ~10 μM (Fig. 3B, dashed line). We observed no adverse effects on the quiescent host cells up to 100 µM, consistent with the fact that I-BET151 in known to be active in tumor cells and cancer cell lines in which c-MYC is overexpressed but not in a variety of other nonimmortalized cell types (18–22).

Using a similar forward genetics approach that identified genetic targets of F0002, *Toxoplasma* tachyzoites were mutagenized with ENU and selected with increasing concentrations of I-BET151. The genomes of three independent I-BET151-resistant (IBETR) mutants were sequenced along with the parental RH strain with coverage of 73 to 100×, and SNP analysis was performed to compare the mutant genotypes to the parental genotype. Among the three independent clones, we identified 19, 8, and 13 SNPs in coding regions, respectively, of which 13 were nonsynonymous. Mutant IBETR-2A8 had an F104L mutation and IBETR-1C6 had an F70C mutation in *TGGT1_254250* (*TgPRELID*). As for the F0002-resistant mutants, the mutations in *TgPRELID* linked to I-BET151 resistance were found within the PRELI domain and not within other regions of the protein (Fig. 2A and C). A third mutant, IBETR-3E6, did not contain a mutation in *TgPRELID* but rather contained a nonsynonymous mutation within the predicted ATPase domain of TGGT1_315560, a predicted transporter protein.

After identifying nonsynonymous *TgPRELID* mutations in two out of three I-BET151-resistant mutants, we obtained DNA from three additional, genetically distinct I-BET151-resistant mutants and sequenced exons 2, 3, and 4 from each. Sequencing results revealed that these three additional I-BET151-resistant mutants also contained nonsynonymous mutations in the PRELI domain of *TgPRELID*. Clones IBETR1-4F10 and IBETR2-5H11 harbored F70S mutations, clone IBETR3-1C6 also contained an F70 mutation to a cysteine, and clones IBETR4-4G12 and IBETR5-2A8 had a mutation at F104 (Fig. 2D), changing a phenylalanine to a leucine or serine. Out of the six independent IBETR mutants, five had mutations in the PRELI domain of *TgPRELID*, and these were found in only two phenylalanine residues of the predicted protein (F70 and F104). This mutation-driven change from a ring-containing amino acid such as phenylalanine to amino acids like serine, cysteine, and leucine is consistent with the amino acid changes identified in the F0002R mutants (Y→C, P→L, and F→S), all of which altered ring-containing (primarily aromatic) amino acids to non-ring-containing residues. Interestingly, mapping of the F0002 resistance and I-BET151 resistance mutations to the predicted structure of the TgPRELID PRELI domain revealed that the mutated residues localize to a distinct, positively charged region of the PRELI domain structure (Fig. 2C).

### I-BET151 mutants are resistant to F0002, and F0002 mutants are resistant to I-BET151.

Given that selection for F0002 and I-BET151 resistance (structures depicted in Fig. 3A) resulted in the generation of resistant parasite lines with similar mutations in the same putative domain of the same gene, we used nonlinear regression to determine the effect of F0002 treatment on I-BET151 mutants and vice versa. All 3 F0002-resistant mutants (originally selected for resistance in 10 μM F0002) challenged with I-BET151 had dose-response profiles significantly different from that of the wild-type parent, and each had a higher MIC that resulted in >50% growth inhibition (MIC_50_) (Fig. 3B; Table 2). We also determined the effect of F0002 on six genetically distinct I-BET151-selected mutants, including five with mutations in TgPRELID and a sixth with a mutation in TGGT1_315560. Three of the I-BET151-selected mutants showed dose-response profiles (*P* < 0.05) significantly different from those of wild-type parasites (Fig. 3C; Table 2), and all 6 mutants showed a trend of having a lower MIC_50_ than the wild-type parent (Table 2). The I-BET151 mutant that showed the greatest resistance to F0002 (as indicated by the curve in Fig. 3C and a MIC_50_ value of 10 µM) was IBETR4-4G12. IBETR4-4G12 and F0002R mutant 3 both harbor F→S mutations in TgPRELID but at amino acid positions 104 and 105, respectively (Fig. 2C and D); these two TgPRELID mutants exhibited the highest degree of cross-resistance (Fig. 3C). These findings indicate that mutations in TgPRELID are associated with parasite resistance to two structurally distinct antiparasitic compounds.

**TABLE 2:** Statistical comparisons between mutant and wild-type *Toxoplasma*^e^

Selection compound^a^/test compound and mutant	IC_50_, µM (SEM)^b^		*P* value (method 3)	MIC_50_ (µM)^c^		*P* value (method 4^d^)
	Wild type	Mutant		Wild type	Mutant	
F3215-0002/I-BET151						
3-R1A2	7.6 (1.04)	328.7 (28.1)	3.1E−06	12.5	None	1.9E−05
1-RB10	14.9 (1.07)	71.6 (1.94)	5.8E−09	25	None	4.7E−10
2-R3C3	12.5 (1.03)	82.1 (1.17)	7.0E−11	12.5	None	0.0060
I-BET151/F3215-0002						
1-4F10	ND^f^	3.17 (1.08)	ND	1.1	3.3	0.12
2-5H11	0.61 (1.07)	1.03 (1.97)	0.85	1.1	3.3	0.062
3-1C6	1.08 (1.15)	2.20 (1.33)	0.54	1.1	3.3	0.448
4-4G12	0.48 (1.19)	5.81 (1.10)	0.011	1.1	10	1.8E−07
5-2A8	0.87 (1.03)	2.59 (1.26)	0.32	1.1	3.3	1.1E−04
6-3E6	0.58 (1.10)	ND	ND	1.1	3.3	1.2E−05

a A 10 µM concentration of F0002 and a 100 µM concentration of I-BET151 were used for selection.

b IC_50_ calculated for use in method 3 (see reference 48 and Materials and Methods) directly comparing IC_50_ values when available for both mutant and wild-type parasites.

c Minimum concentration that resulted in >50% growth inhibition compared to the untreated controls.

d *P* value comparing entire dose-response curves for each mutant to the wild-type parent using method 4 (see reference 48 and Materials and Methods). For each mutant, a separate wild-type assay was conducted in parallel.

e IBETR mutants were challenged with F0002, and F0002-resistant mutants were challenged with I-BET151.

f ND, not determined.

### *Toxoplasma* F0002-resistant mutants generated via CRISPR/CAS9 have a variety of mutations near the CAS9 cut site in TgPRELID.

We attempted to edit the *T. gondii* genome at the TgPRELID locus using CRISPR (clustered regularly interspaced short palindromic repeat)/CAS9. Parasites were transfected with a plasmid encoding the CAS9 nuclease and a guide RNA (gRNA) targeting exon 4 of *TgPRELID*, along with a cloned homology repair sequence taken from exon 4 of F0002-resistant mutant 3 (F105S) (Fig. 2C and D). We isolated populations of parasites from 2 separate transfections that grew in 5 μM F0002. We cloned one of these populations by limiting dilution in 5 μM F0002 and then directly compared the resistance phenotype of 2 clones to the wild-type parental line (RH) by exposure to 10 μM F0002 for 24 h. As expected, 10 μM F0002 significantly reduced parasite replication in wild-type (WT) RH *T. gondii* (*P* < 0.05) (Fig. 3D), while CR mutant clones SOL1 and SOL2 (CRSOL1 and CRSOL2, respectively) were F0002 resistant (*P* > 0.05, comparing vehicle treatment to F0002) (Fig. 3D). We also tested I-BET151 resistance in the CR-derived mutant clones. In contrast to the 3 ENU-derived F0002-resistant mutants which were highly resistant to I-BET151 at all tested doses (including 50 μM [Fig. 3B]), CR mutant clones SOL1 and SOL2 were both as susceptible to 50 μM I-BET151 as the wild-type parental line (RH [Fig. 3E]).

To identify the mutations driving F0002 resistance in our CR mutant populations and in clones SOL1 and SOL2, we sequenced *TgPRELID* exons 2, 3, and 4 in the mutant populations. As expected, we did not observe any mutations in exons 2 and 3. Unexpectedly, however, we found that neither of the 2 independently derived mutant populations, nor the isolated clones, used the homology repair template (harboring the F0002R3-R1A2 mutation [Fig. 2D]) as evidenced by having a wild-type codon for F105. Instead, we found that both mutant populations had the same 3-nucleotide deletion of valine 143, which kept the *TgPRELID* gene in frame (Fig. S2A), and both populations appeared to harbor parasites that acquired this deletion in two distinct ways (outlined in Fig. S2A and B): by deletion of AGG (F0002-CR1) or by deletion of GTT (F0002-CR2) (Fig. 2D and S2B). When we sequenced isolated clones CRSOL1 and CRSOL2, we found that these strains harbored distinct mutations near the CAS9 cut site but ones which still led to a mutation in valine 133, in this case to a phenylalanine (V133F) (Fig. 2D and S2B). These data further implicate TgPRELID mutations in F0002 resistance, expand the number of possible TgPRELID mutations that are associated with F0002 resistance (in this case, valine 133), and represent the first mutation thus far identified that is linked only to F0002, and not I-BET151, resistance.

10.1128/mSphere.00229-16.2 FIG S2  (A) Schematic of the CRISPR/CAS9 targeted region (top and bottom strand) of TgPRELID showing the PAM sequence (underlined) and the bound guide RNA that was expressed in wild-type *T. gondii* parasites along with a homology repair template flanking the cut site (shown as an orange dotted line). The corresponding amino acid sequence is shown on the last row. (B) Raw sequencing data from wild type (top), CRISPR/CAS9-derived mutant populations (middle), and mutant clones CRSOL1 and -2 (bottom) showing the possible ways that the locus was mutated during CAS9 cleavage and nonhomologous end joining (based on these data, we have no evidence that the CRISPR-derived F0002-resistant mutants incorporated the homology repair template with a mutant form of TgPRELID from F0002-resistant mutant 3 [R1A2]). Based on PCR-based sequencing of the mutant populations, CRISPR and subsequent repair deleted either GGT (deletion 1) or AGG (deletion 2), which deletes valine 133 and either maintains K132 (deletion 1) or converts it to a methionine (deletion 2). For clones CRSOL1 and CRSOL2, the mutation could have emerged by deletion of “GGTG” followed by repair with “GTTT,” leading to two point mutations resulting in the conversion of valine 133 to phenylalanine. Other intermediates are also possible, but both sets of mutants implicate valine 133 in F0002 binding and/or function. Download FIG S2, DOCX file, 0.6 MB. Copyright © 2017 Jeffers et al. 2017 Jeffers et al. This content is distributed under the terms of the Creative Commons Attribution 4.0 International license.

### TgPRELID localizes to the parasite mitochondrion.

**FIG 4:**
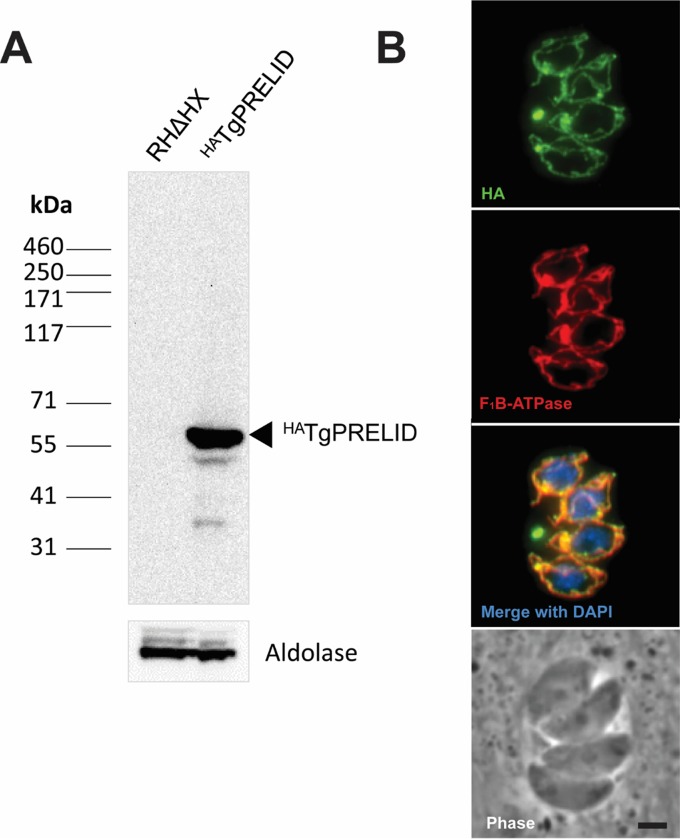
Ectopic expression of ^HA^TgPRELID. (A) Western blot assay using anti-HA antibody against ^HA^TgPRELID-expressing parasites shows a band at the predicted molecular mass of 55 kDa that is absent in parental RHΔHX parasites. TgAldolase was probed as a loading control. (B) IFA of the ^HA^TgPRELID expressed in tachyzoites shows colocalization with the mitochondrial protein F_1_B-ATPase. Bar, 2 µm.

As no PRELI domain protein has been characterized in apicomplexan parasites to date, we determined the cellular localization of TgPRELID. We attempted to add a C-terminal 3× hemagglutinin (HA) tag at the endogenous TgPRELID locus. PCR analysis of a number of clones verified the correct integration of the tagging vector; however, immunofluorescence assays (IFAs) of these clones did not detect any antibody staining above background (Fig. S3), presumably due to low abundance of the protein or the C terminus being incorrectly predicted in ToxoDB. Similar negative results were obtained when we expressed a C-terminally tagged *TgPRELID* cDNA-derived minigene from one of the F0002-resistant mutants (F0002R3-R1A2) off a highly active *gra1* promoter (data not shown). However, when this same cDNA TgPRELID minigene was fused to an N-terminal HA tag and ectopically expressed from the *gra1* promoter, we were able to detect protein. Western blotting detected a single band at the predicted size of ~54 kDa, and an IFA revealed localization to the parasite’s mitochondrion (Fig. 4). Although there was no mitochondrial targeting sequence predicted by either TPpred2 (23) or TargetP 1.1 (24), the mitochondrial localization of TgPRELID is consistent with the localization of homologous human and yeast PRELI domain-containing proteins in the intermembrane mitochondrial space (11, 12). We think it unlikely that the single point mutation in TgPRELID from F0002R3-R1A2 is responsible for the observed mitochondrial localization, although this can be tested in future experiments. Unfortunately, attempts to clone out clones stably expressing N-terminally tagged TgPRELID failed, suggesting that it might ultimately be toxic when expressed at the high levels necessary to detect it in our assays.

10.1128/mSphere.00229-16.3 FIG S3  Endogenous tagging of TGGT1_254250/TgPRELID. (A) Strategy for replacement of the endogenous coding sequence with sequence encoding a C-terminal triple HA tag by single-crossover homologous recombination, with locations of primers A, B, and C used for screening. (B) PCR analysis of five clones for correct integration of the tagging construct using primers A and B to amplify the unmodified parental locus and primers A and C to amplify the intended integrated locus. Three correct clones were identified (clones 1, 2, and 5, marked with asterisks). P, parental. (C) Immunofluorescence analysis of the correctly integrated clones did not detect any signal over that seen in the parental parasites. Images depict clone 1, which is representative of all three correctly integrated clones. Download FIG S3, DOCX file, 0.4 MB. Copyright © 2017 Jeffers et al. 2017 Jeffers et al. This content is distributed under the terms of the Creative Commons Attribution 4.0 International license.

## DISCUSSION

In this report, we describe the use of forward genetics to investigate mechanisms of action for two novel antiparasitic compounds. Our findings identified a single gene that is capable of conferring mutation-driven resistance to two structurally diverse compounds. There are multiple mechanisms for how this might occur. Under one scenario, F0002 and I-BET151 may work by targeting the same site(s) in TgPRELID, and therefore, similar mutations confer cross-resistance to both inhibitory molecules. Our structural modeling of the localization of the identified mutations provides support for this potential mechanism of resistance, as the mutations cluster within a specific region of the PRELI domain. Additionally, while the two compounds are structurally distinct, they share drug-like properties and are similarly sized (Fig. 3A), suggesting that they could theoretically interact with the same binding site. Another possibility is that F0002 and I-BET151 are inactivated and/or sequestered by mutant forms of TgPRELID but that the actual target(s) of each of these compounds is distinct. Inactivation of the compounds could occur via enzymatic attack (e.g., beta-lactamases) or through a chemical pump (e.g., chloroquine resistance in *Plasmodium falciparum* [25]). This scenario would implicate TgPRELID as a multidrug resistance protein. It will be of interest to determine if mutations in this gene also confer resistance to other compounds with antiparasitic activity, which would provide further support to this model of TgPRELID-mediated resistance. Further studies (particularly structural) will help to shed light on which of these scenarios is most likely.

Our finding that a mutation of the putative ABC transporter TGGT1_315560 also mediates resistance to both compounds suggests that they have, at minimum, a common site of action. We predict that the mutation of the ATPase domain of the transporter might alter the substrate-binding properties of the channel to promote pumping of the active compounds away from their target.

It is surprising that mutant TgPRELID mediates resistance to I-BET151, considering that at least one of the mutant forms of TgPRELID has very clear localization to the mitochondrion. The bromodomain target(s) of I-BET151 is expected to be a regulator of gene expression and localized to the nucleus, so it is unclear how a mitochondrial protein might interfere with I-BET151 action. A proteomic analysis of global lysine acetylation in *Toxoplasma* has identified a substantial proportion of proteins with acetylated lysines that are localized to the mitochondrion (14); therefore, it remains possible that a bromodomain protein may also localize to this organelle.

The endogenous function of TgPRELID remains unknown and may be challenging to dissect. Interestingly, in a recently published CRISPR/CAS9 screen of all predicted genes in *T. gondii*, TgPRELID had a phenotype score of −4.59, meaning that parasites harboring plasmids encoding guide RNAs (gRNAs) targeting TgPRELID were strongly selected against during *in vitro* growth (by ~25-fold [26]). These data indicating strong selection for TgPRELID expression during tachyzoite growth *in vitro* suggest that *TgPRELID* encodes a putative essential gene and may also explain why both CRISPR-generated F0002-resistant populations had exactly the same sets of 2 deletions and/or nonhomologous end-joining (NHEJ)-mediated repairs. In each case, we identified a 3-nucleotide deletion that preserved the reading frame and deleted K132 (see Fig. S2 in the supplemental material), which is remarkable given the large number of possible NHEJ-mediated repair events that could have occurred. It is likely that selection was operating on the CAS9-cleaved gene to preserve the function of TgPRELID given that parasites with disrupted TgPRELID would be rapidly outcompeted. A similar explanation could be made for the isolated clones SOL1 and SOL2, where after deletion of the GGT near the PAM site a multistep repair and NHEJ event would be required to generate the GTG→TTT mutant while preserving the reading frame (see Fig. S2B). These ideas could be tested by targeting other sites in the gene for editing and examining the resulting NHEJ events.

Initial attempts to endogenously tag the C terminus resulted in integration at the locus as expected but very little, if any, detectable tagged protein. We also tried to overexpress a C-terminally tagged mutant TgPRELID (from F0002R3-R1A2) driven by the *gra1* promoter and also failed to detect any tagged protein. It was only after adding the HA tag on the N terminus of this same *TgPRELID* mutant form that we were able to detect TgPRELID protein, although parasites rapidly lost the TgPRELID cassette very rapidly during drug selection. As mentioned above, the localization of the endogenous, wild-type TgPRELID protein will be necessary to fully confirm its mitochondrial localization, although we do not expect that the point mutation in the construct that we used for these localization studies played a significant role in determining the localization. While it is possible that the C terminus is incorrectly predicted in ToxoDB, unusual features at the C-terminal end of TgPRELID may explain why it is not amenable to C-terminal epitope tagging. As shown in Fig. 2B, the C terminus is predicted to be highly disordered. It is possible that tagging the C terminus blocks key interactions between the disordered C terminus and unknown proteins that stabilize TgPRELID. Trafficking of the protein to the mitochondrion may also result in cleavage of a portion of the C terminus, or the putative transmembrane (TM) could indicate that the protein is a mitochondrial tail-anchored protein and that a portion of the C terminus is cleaved during or after membrane insertion. Another possible explanation is that TgPRELID protein turns over very rapidly (or is expressed at very low levels) *in vivo* and is reliably detectable only using overexpression constructs. This could be tested further by expressing N-terminally tagged TgPRELID off its cognate promoter. Interestingly, this disordered C terminus is present in all identified coccidian sequences but is absent in all of the identified *Plasmodium* sequences (Fig. S1A). Given these structural differences between coccidian parasites and *Plasmodium* spp., it is possible that these proteins have distinct functions in these two parasite lineages. This idea finds some support in the way in which the sequences cluster on the neighbor-joining tree (Fig. S1B).

PRELI domain-containing proteins in humans and yeast have been implicated in transfer of phospholipids across mitochondrial membranes to maintain the appropriate lipid composition of the mitochondrial membrane. Humans and yeast have four PRELI domain-containing proteins, while *Toxoplasma* and other apicomplexans that we have examined appear to contain a single PRELI domain-containing protein. BLAST analysis suggests that the parasite protein is most similar to PRELID3B (or SLMO2) in humans; however, the sequences are so divergent that it is difficult to confidently predict which homologues are functionally related.

PRELID1 in humans mediates transfer of phosphatidic acid (PA) to the inner mitochondrial membrane. PA is a precursor for cardiolipin (CL) synthesis, which is necessary for mitochondrial structure and integrity. Knockout of PRELID1 results in a reduction of CL in the mitochondrial membranes, leading to release of cytochrome *c* and promotion of proapoptotic pathways in the cell (27). PRELID1 is inherently unstable but is stabilized by interaction with TRIAP1, allowing PA transfer to the inner mitochondrial membrane and maintenance of CL levels (28). The only other PRELI domain-containing homologue that has been characterized, PRELID3B (also known as SLMO2 in humans), also regulates the transfer of phospholipids to the inner mitochondrial membrane but has a substrate preference for phosphatidylserine (PS), contributing to phosphatidylethanolamine synthesis and maintenance of mitochondrial integrity (29). The structures of both human and yeast homologues PRELID1 and Ups1 consist of a hydrophilic pocket and hydrophobic patches to promote binding to the phospho head group and fatty acid chain of PA, respectively (12, 30). Mutation of another cluster of hydrophobic residues on PRELID1 and Ups1 also disrupted TRIAP1 or Mdm35 (the yeast homologue) association (12, 30). It is possible that the *TgPRELID* mutations observed in the resistant lines generated in this study deregulate phospholipid transfer into the mitochondrion, subsequently altering mitochondrial membrane composition. How this might affect the parasite response to specific compounds remains to be determined in future studies. Moreover, it is of interest to determine if *TgPRELID* is a gene that is poised to mediate resistance to a variety of compounds, which could have broad implications for apicomplexan parasite drug development. These studies also highlight the utility of forward genetics and sequencing to elucidate the potential mechanisms of action of antiparasitic compounds that are obtained through high-throughput compound library screening and alert drug development efforts toward any lead compounds that parasites may be poised to quickly develop resistance.

## MATERIALS AND METHODS

### Parasite strains and cell culture.

Monolayers of human foreskin fibroblasts (HFFs) were cultured and grown as described previously (1, 6). *Toxoplasma* type I RH strain parasites were used in all experiments. For some ethylnitrosourea (ENU) mutagenesis experiments and luciferase-based growth assays, we used a wild-type RH strain previously engineered to express click beetle luciferase under the dihydrofolate reductase (DHFR) promoter and the fluorescent protein dsRED under control of the GRA1 promoter (1).

### Ethylnitrosourea mutagenesis.

Parasites were allowed to infect confluent HFF monolayers for 16 to 18 h in complete Dulbecco's modified Eagle medium (cDMEM) (DMEM, 10% fetal calf serum [FCS]). Infected monolayers were washed with DMEM containing 0.1% FCS and then treated for 2 h with freshly made 250-mg/ml ENU in 0.1% FCS-DMEM. Monolayers were washed one to two times with fresh cDMEM and then allowed to incubate overnight. Flasks were scraped, and parasites were released by needle passage. Following centrifugation, parasites were used to infect a T-75 (75-cm^2^) flask containing confluent HFFs in the presence of 10 μM F3215-0002 (Life Chemical Corporation) and allowed to grow for at least 5 days. Plaques were identified by microscopy, and drug-resistant clones were obtained by limiting dilution in cDMEM containing the same concentration of drug used for initial selection. To generate I-BET151-resistant parasites, ENU-mutagenized populations were divided into 24-well plates and passaged at least 5 times in the presence of 5 µM I-BET151 (GlaxoSmithKline, United Kingdom) and then in I-BET151 concentrations incrementally increasing with every second passage by 1 µM until parasite populations could grow in 10 µM compound. Independent I-BET151-resistant populations were cloned using limiting dilution.

### Next-generation sequencing, candidate SNP identification, and validation.

DNA was isolated from 3 genetically distinct F0002-resistant clones and the parental wild-type strain using the DNAzol reagent according to the manufacturer’s instructions. Next-generation sequencing libraries were constructed using the Illumina TruSeq DNA kit. Bar-coded samples were sequenced on a single lane of an Illumina Genome Analyzer, generating a total of 283 million 50-bp paired-end reads. Reads were aligned to the *Toxoplasma* genome (strain GT1; v7.0) using Bowtie 0.12.7 (30). Default settings were used except that the maximum number of mismatches in the seed sequence was set to 3 (-n 3 in Bowtie options). Alignments were analyzed using the mpileup command from SAMtools (31) and a custom Perl script. Candidate SNPs for each mutant were identified at sites where coverage was >3×, 70% of the reads agreed with the SNP, and the SNP was not present in wild-type RH sequence data. The script also used the v7.0 GT1 genome annotation to determine if the SNP was present in a coding region and the type of polymorphisms (synonymous, nonsynonymous, nonsense, or missense). To validate candidate SNPs, the following PCR primers were generated flanking the SNP and the reverse primer encoded an M13 promoter primer binding site: Exon 2 For, GTAGAGGAGGTAGAAGTCGAGACA; Exon 2 Rev_M13R, CAGGAAACAGCTATGACCTTATCGGTCAAAAGCTAAAGGAG; Exon 3 For_M13F, TGTAAAACGACGGCCAGTTCTTCTTCTCTTGAACAAATCGTG; Exon 3 Rev, CTCACTGTGGCATGTTCCTGGAT; Exon 4 For_M13F, TGTAAAACGACGGCCAGTTGAGAGAAACGAAGAATCAGAATG; and Exon 4 Rev, CGAAATGCCGCACCGGCACAACGC (M13R or M13F sequences are underlined). The PCR products were purified using a PCR product cleanup column (Qiagen), sequenced using Sanger sequencing, and then compared to the multiple versions of the GT1 genome using the *Toxoplasma* genome database.

Genomic DNA from three independent I-BET151-resistant clones, capable of growth in at least 100 µM I-BET151, was isolated using the Qiagen DNeasy Blood and Tissue kit. Sequencing libraries were prepared for each sample using the Illumina TruSeq DNA kit. Bar-coded samples were sequenced on a single lane on an Illumina Genome Analyzer, generating over 411 million 100-bp paired-end reads. The reads were aligned to the *Toxoplasma* genome (strain GT1; v8.0) using CLC Genomics Workbench v5.1.5 with default parameters. SNP analysis was performed with CLC Genomics Workbench v5.1.5, and candidate SNPs in the three resistant mutants were manually curated to identify SNPs that were not present in the parental RH strain and to confirm that 75% of the reads agreed with the SNP.

### Sequence analyses.

The domains in TgPRELID were validated using the publicly available Pfam server (32), and TMPred (33) was used to identify putative transmembrane domains. Disopred3 (34) was used to predict protein tertiary structure in relation to resistance-linked mutations and to further inform tagging experiments. Disopred3 calculates a likelihood of both protein disorder and protein binding across the query protein based on existing structures found in public databases. Orthologues of TgPRELID were identified by orthology and BLASTP on ToxoDB and PlasmoDB. Multiple sequence alignments were generated in Clustal Omega (35) and visualized in Jalview (36).

### CRISPR/CAS9 genome editing.

We scanned the *TgPRELID* gene for PAM sequences near mutations linked to F0002 resistance and identified the sequence ACAGCCACCTACAAGGTGTTTGG (PAM sequence underlined), which starts 68 bp downstream of the exon 4 mutation found in strain RHF0002R-2. We introduced this guide RNA (gRNA) sequence downstream of the U6 promoter in the plasmid pSAG1-CAS9-U6-sgUPRT (kindly provided by David Sibley, Washington University in St. Louis [37]) using the Q5 site-directed mutagenesis kit. Specifically, pSAG1-CAS9-U6-sgUPRT was used as a PCR template with forward primer ACAGCCACCTACAAGGTGTTGTTTTAGAGCTAGAAATAGC (gRNA sequence underlined) and reverse primer AACTTGACATCCCCATTTAC, and the resulting product (pSAG1-CAS9-U6-sgTgPRELID) was transformed into *Escherichia coli*. To generate the repair template, exon 4 was amplified from genomic DNA isolated from the F0002R3-R1A2 F0002-resistant mutant using Exon 4 F/R primers (described above) and Topo cloned into the PCR2.1 Topo vector (Invitrogen). The resulting construct (PCR2.1_TgPRELID_Exon4) was verified by sequencing. Approximately 2 × 10^7^ RH:dsRED:LUC parasites were transfected with 20 μg of pSAG1-CAS9-U6-sgTgPRELID and 50 μg of PCR2.1_TgPRELID_Exon4, allowed to grow in HFFs for ~18 h, and then placed under F0002 selection (5 μM). Parasites were passaged a minimum of every 5 days. Two transfections were performed in parallel, and we isolated two genetically distinct F0002-resistant populations and sequenced PCR products derived from exons 2, 3, and 4 of the *TgPRELID* gene using the same approach that was used to sequence the loci of ENU-derived mutants. We cloned one of these populations by limiting dilution and isolated 2 parasite clones (CRSOL1 and CRSOL2). In this case, we cloned the exon 4 PCR products into PCR2.1 Topo and sequenced 2 individual colonies derived from each clone. To quantify their relative resistance to F0002 and I-BET151, CRSOL1 and -2 were each grown in 10 μM F0002 and 50 μM I-BET151 for 24 h, and then parasite numbers were determined using direct counting with a hemacytometer and their growth in the presence of drug was compared to that of the wild-type parental RH strain.

### Endogenous tagging and ectopic expression of TgPRELID.

A triple HA tag was added to the C terminus of TgPRELID using the pLIC-3×HA vector as previously described (38). Plasmid pLIC-254250^3×HA^ was generated by using primers 5′ACGGGAATTCCCTAGGGTGAGAAACATTTGCGCATGC3′ and 5′CGTACGGGTACCTAGGCGAGGGGGGTCGTCGAC3′ to amplify the 3′ genomic region of *TgPRELID* for cloning into the AvrII site of pLIC-3×HA by InFusion ligase-free cloning (Clontech). Primers 5′ATCTTCGTTTCTCTCACGC3′ and 5′ATCAGAATGTGAGGCGAAG3′ were used in conjunction with the Q5 mutagenesis kit (New England BioLabs) to insert an adenosine base within intron 4, generating an EcoRV site for linearization of the tagging vector prior to transfection. Transfectants were selected by three rounds of pyrimethamine treatment before cloning by limiting dilution. Clones were screened for correct integration events with the primers A (5′CCTGGGAGCAGCGATCAATCG3′), B (5′CGTTTCTGCACCAAGTTCTACTTG3′), and C (5′CGTGTGTTACGTTTACTAACG3′) (see Fig. S1 in the supplemental material).

For ectopic expression of TgPRELID, we used forward primer 5′-**GGGGACAAGTTTGTACAAAAAAGCAGGCT**ATGTACCCGTACGACGTCCCGGACTACGCGAGACTCTTCGAGAAGACGTTCGTC (B1 site in bold, HA tag underlined) and reverse primer 5′-**GGGGACCACTTTGTACAAGAAAGCTGGGT**CTACGAGGGGGGTCGTCGACTTCT (B2 recombination site in bold). For these studies, F0002-resistant RH strain 3^R1A2^ RNA was used in cDNA synthesis reactions with Superscript II reverse transcriptase (Invitrogen, Carlsbad, CA), and the resulting cDNA (harboring the 3^R1A2^ mutation) was used as a template in PCRs. Gel-purified fragments were cloned using BP Clonase into the pDONR221 entry vector followed by LR Clonase into the pGRA1-ATT-GRA2 destination vector. All constructs were verified by sequencing. Parasites were transiently transfected with 50 μg of the construct and fixed at 18 h posttransfection. Parasites were grown in normal cDMEM or in cDMEM plus 10 μM F0002 as indicated and stained with rat monoclonal anti-HA antibody as described below.

### Luciferase-based parasite growth assays.

Parasites were allowed to infect confluent monolayers of HFFs grown in 96-well plates (10,000 parasites/well) in cDMEM. At the end of the growth period, wells were washed once with 100 µl of phosphate-buffered saline (PBS) and harvested by adding 100 µl of 1× cell culture lysis reagent (Promega, Madison, WI) to each well. Fifty microliters of each sample was added to a white-bottom 96-well plate and mixed with 20 µl of freshly thawed luciferase assay reagent (LAR; Promega, Madison, WI). Luciferase activity was measured using a Centro XS^3^ LB960 luminometer (Berthold Technologies, Oak Ridge, TN) within 2 min of adding the LAR to each well (10-s measuring time per well).

### Calcium ionophore-based growth assays.

To confirm results with luciferase-based growth assays and to measure parasite growth when parasite strains did not express the luciferase gene, we adapted usage of the calcium ionophore A23187 (39) to induce egress so that we could more readily count parasites emerging from infected cells after growth in different drug treatment regimens. Specifically, we infected 24-well plates of confluent HFFs with 32,400 parasites (estimated multiplicity of infection [MOI] of 0.3). Parasites were allowed to invade overnight (up to 18 h), and then the appropriate drug treatments were applied. In order to quantify parasite growth at the end of the treatment period, the medium was gently aspirated and replaced with 1.5 ml of 10 μM A23187 for 5 min (39). Wells were observed visually to ensure that egress was occurring and complete. One milliliter was removed from each well and centrifuged at 800 × *g* for 10 min. After aspiration of exactly 750 μl of medium, parasites were resuspended in the remaining medium and 10 μl of this resuspension was counted on a hemocytometer. Similar assays were also conducted, but by using syringe lysis rather than ionophore treatment, to quantify the growth of the CRISPR-generated mutants in F3215-0002 and I-BET151.

### Structure prediction and visualization.

The I-TASSER server (40) was used to generate a model of TgPRELID (residues 1 to 162), using human SLMO1 (PDB code 4XZV:B [12]) as a template for threading. With the exception of template specification, all other settings were used at their default values. Visualization of the predicted TgPRELID structure and mapping of electrostatic potential were performed using the UCSF Chimera software package (41) and the tools PDB2PQR (42, 43) and APBS (44).

### Immunofluorescence assays.

Localization of tagged TgPRELID was determined by immunofluorescence staining, as described previously (17). Confluent HFF monolayers grown on coverslips were infected with tachyzoites for 18 h, and then infected monolayers were fixed in 4% paraformaldehyde, permeabilized with 0.2% Triton X-100 in PBS, and blocked in 3% bovine serum albumin (BSA). Fixed monolayers were incubated with primary antibody diluted in 3% BSA-PBS for 1 h at room temperature, washed in PBS (three 15-min washes), and then stained with fluorophore-linked secondary antibody for 1 h, followed by washing in PBS (three 15-min washes). Cells were stained with 4′,6-diamidino-2-phenylindole (DAPI) prior to mounting in Vectashield antifade solution. Antibodies used were anti-HA (1:2,000; Roche), anti-F1B ATPase (1:4,000; a gift from Peter Bradley), and anti-rat-488 (1:2,000) and anti-mouse (1:2,000) (both from Molecular Probes).

### Western blotting.

Parasite lysates were prepared from infected HFF monolayers that were scraped, washed in PBS, resuspended in parasite lysis buffer (150 mM NaCl, 50 mM Tris-Cl, pH 7.5, 0.1% NP-40) supplemented with mammalian protease inhibitor cocktail (Roche), and sonicated. The insoluble fraction was cleared by centrifugation, and protein concentration was quantified using the Bio-Rad DC assay. Samples were suspended in SDS-PAGE buffer with beta-mercaptoethanol before being loaded on a 4 to 12% Bis-Tris Novex gradient gel and run in morpholinepropanesulfonic acid (MOPS) running buffer. Separated protein was transferred to nitrocellulose membrane and blocked in 4% milk–Tris-buffered saline–Tween (TBST) for 1 h at room temperature followed by incubation with anti-HA (1:2,000; Roche) in 4% milk-TBST, washing in TBST, and anti-rat-horseradish peroxidase (HRP; 1:2,000; GE Healthcare) incubation in 4% milk-TBST. After imaging, blots were then stripped using Restore stripping buffer (Thermo Fisher Scientific) before incubation with anti-PfAldolase-HRP (1:2,000; Abcam), followed by washing in TBST and imaging. Chemiluminescent imaging of membranes was performed using Pierce enhanced chemiluminescence (ECL) Western blotting substrate and a Protein Simple chemiluminescent imager.

### Statistical analyses.

To compare mutant and wild-type drug responsiveness to 10 μM F0002 or 100 μM I-BET151, we performed 2-way analysis of variance (ANOVA) and *ad hoc t* tests for each parasite clone/population in the presence or absence of drug. Type I error rate was controlled using the method of Holm (45–47), and differences were deemed significant at a *P* value of <0.05.

Growth curves between mutant and wild-type parasites were generated and analyzed using GraphPad Prism using methods 3 and 4 as outlined in reference 48. These methods were chosen because each mutant was assayed only once at each dose. Method 3 allows for a direct test of differences in IC_50_s obtained in mutant and wild-type lines, while method 4 is more sensitive to overall changes in the dose-response profile (without the need to estimate IC_50_ values or other parameters [48]). To do this, each F3215-0002-resistant mutant was tested for growth in various concentrations of I-BET151, and each I-BET151 mutant was tested for growth in various concentrations of F3215-0002. Each mutant was assayed separately along with the parental wild-type strain (therefore, each assay had its own wild-type control). Data from mutant and wild-type strains were converted to percent growth compared to vehicle-only controls, and nonlinear regression curves were generated for each mutant along with its corresponding wild-type assay using the log_10_ of the compound dose. Curves were generated allowing for a variable slope but constrained to be between 0 and 100 [“log(inhibitor) versus normalized response—variable slope”]. For method 3, if the IC_50_ value could be estimated for both the wild type and the mutant, the IC_50_ values were compared using a simple *t* test (using the IC_50_ value, standard error, and sample size). For method 4, the same nonlinear regression curves as for method 3 were used, and the sum of the sum of squares for the wild-type and mutant data curves analyzed separately was compared to the sum of squares when the data from wild-type and mutant strains were combined (again, as outlined in reference 48). This method compares the entire dose-response curve and does not estimate or compare IC_50_ values. We determined an *F* statistic using the following equation: *F* = [(SS_combined_ − SS_separate_)/(DF_combined_ − DF_separate_)]/(SS_separate_/DF_separate_). *P* values were calculated using the standard *F* distribution and deemed significant at a *P* value of <0.05. These data are represented in Table 2.

### Accession number(s).

The raw genome sequence data for F0002 and I-BET151 mutants (and their corresponding parental lines) have been submitted to the NCBI Short Read Archive under BioProject no. PRJNA356224.
