# Supplementary figures and images for: TgPRELID, a Mitochondrial Protein Linked to Multidrug Resistance in the Parasite Toxoplasma gondii
Source: mSphere. 2017 Feb 1;2(1):e00229-16. doi: 10.1128/mSphere.00229-16 (PMC5288566; doi:10.1128/mSphere.00229-16)

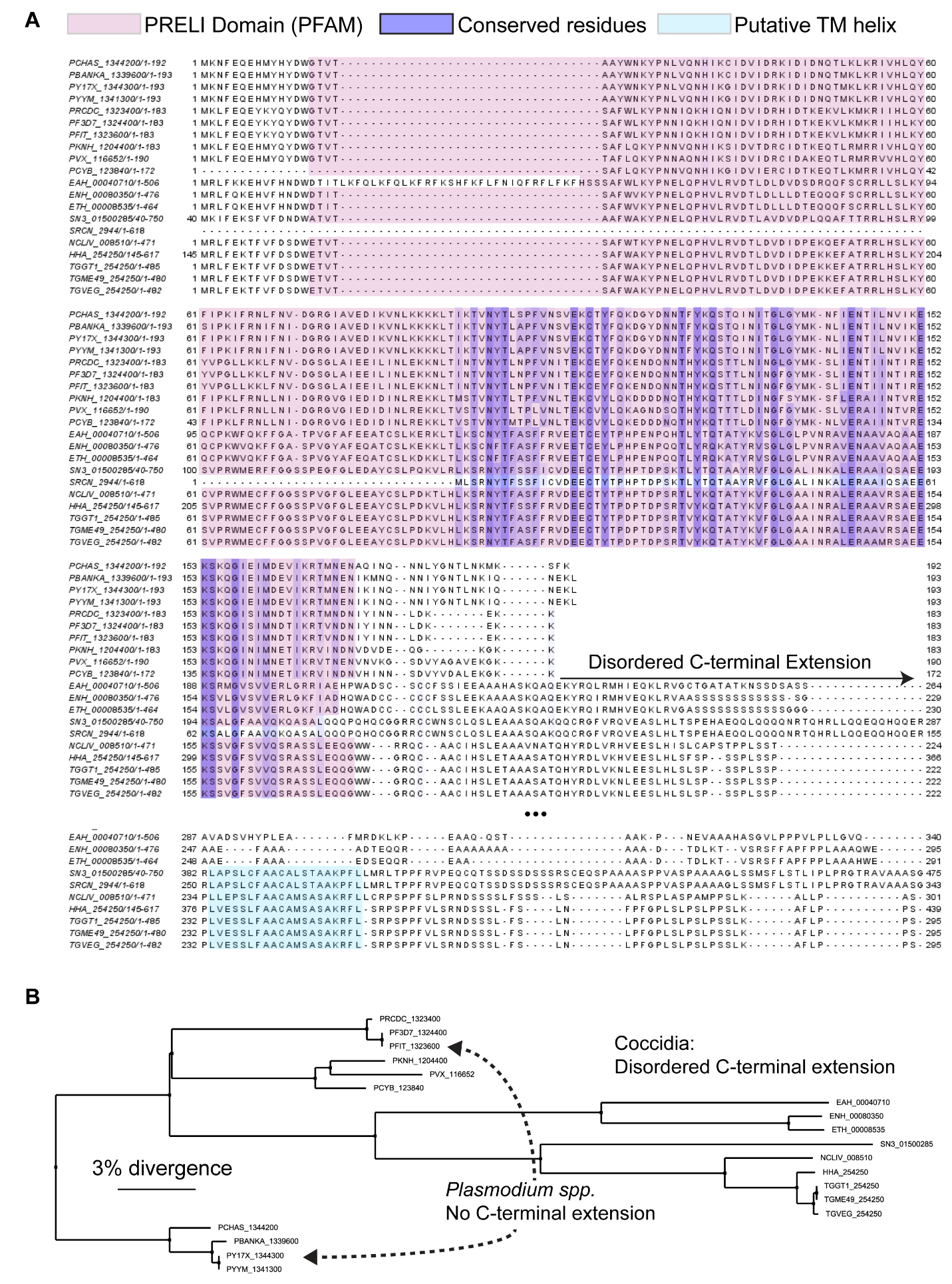

Supplement: FIG S1 [file sph001172228sf1.docx]

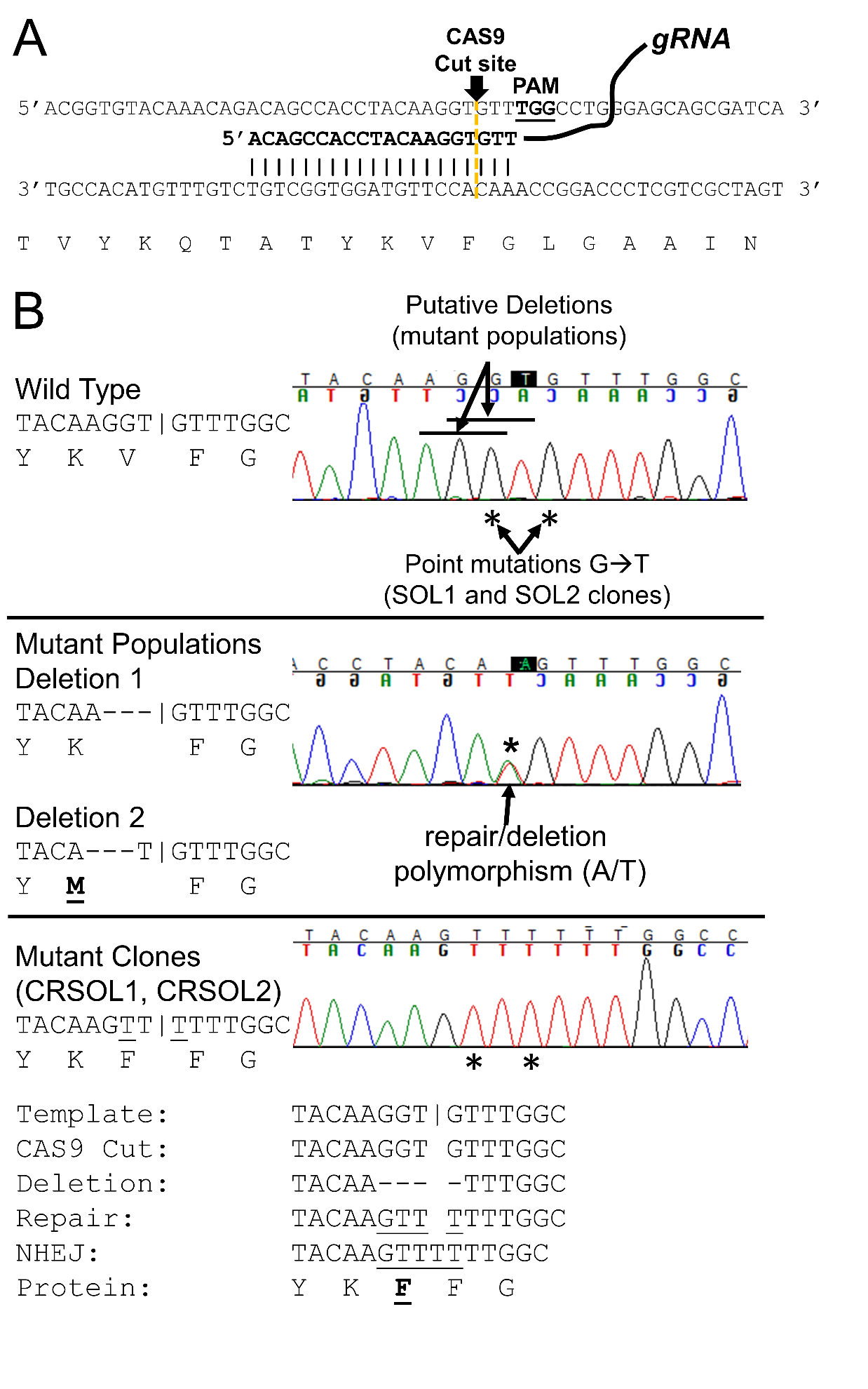

Supplement: FIG S2 [file sph001172228sf2.docx]

**
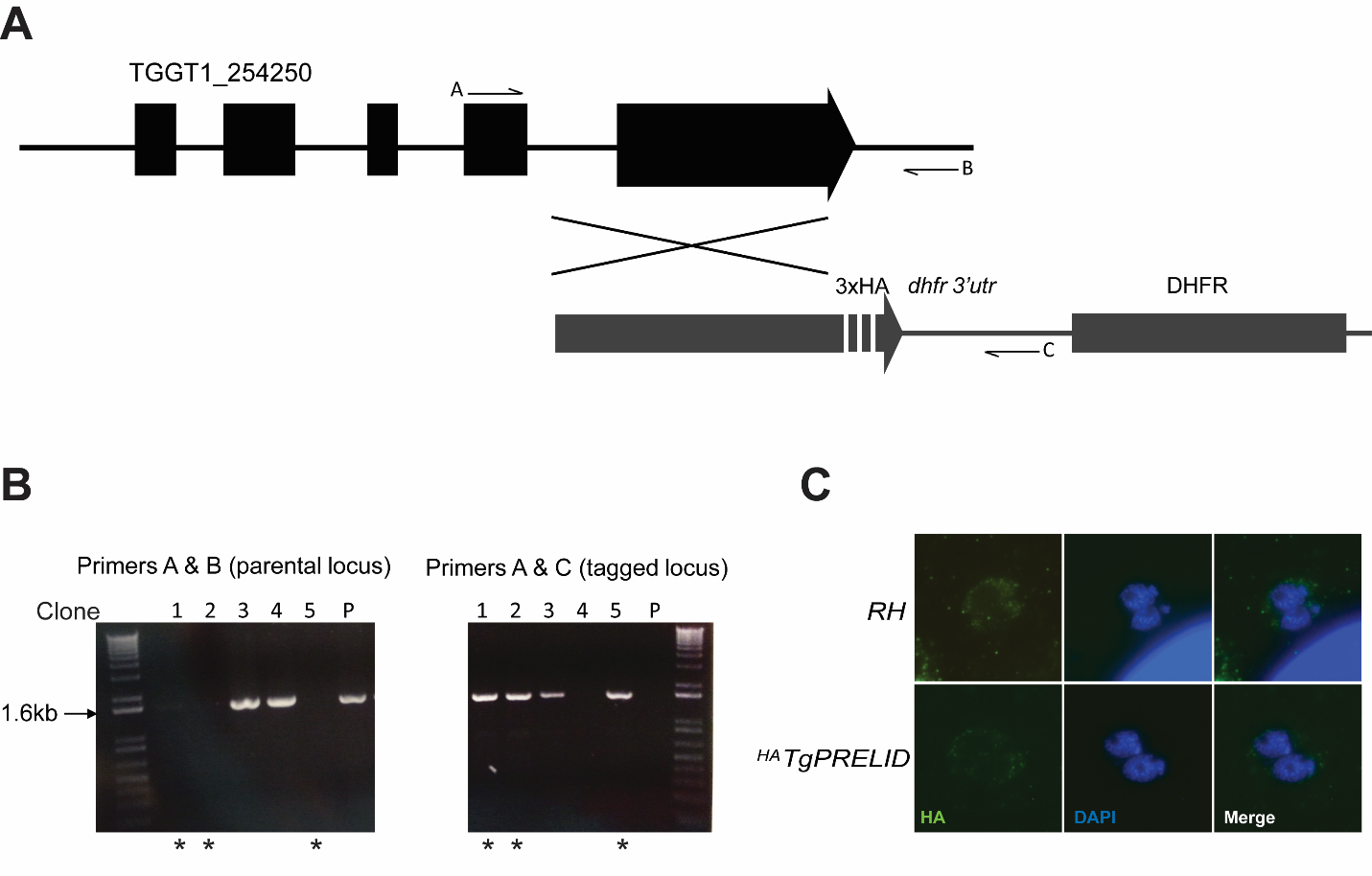
**

Supplement: FIG S3 [file sph001172228sf3.docx]
